# The future of evolutionary medicine: sparking innovation in biomedicine and public health

**DOI:** 10.3389/fsci.2023.997136

**Published:** 2023-02-28

**Authors:** B. Natterson-Horowitz, Athena Aktipis, Molly Fox, Peter D. Gluckman, Felicia M. Low, Ruth Mace, Andrew Read, Paul E. Turner, Daniel T. Blumstein

**Affiliations:** 1 Division of Cardiology, David Geffen School of Medicine, University of California, Los Angeles, Los Angeles, CA, United States; 2 Department of Human Evolutionary Biology, Harvard University, Cambridge, MA, United States; 3 Department of Psychology, Arizona State University, Tempe, AZ, United States; 4 Center for Evolution and Medicine, Arizona State University, Tempe, AZ, United States; 5 Department of Anthropology, University of California, Los Angeles, Los Angeles, CA, United States; 6 Department of Psychiatry and Biobehavioral Sciences, Semel Institute for Neuroscience and Human Behavior, University of California, Los Angeles, Los Angeles, CA, United States; 7 Koi Tū: The Centre for Informed Futures, University of Auckland, Auckland, New Zealand; 8 Liggins Institute, University of Auckland, Auckland, New Zealand; 9 Department of Anthropology, University College London, London, United Kingdom; 10 Center for Infectious Disease Dynamics, Department of Biology, The Pennsylvania State University, State College, PA, United States; 11 Department of Entomology, The Pennsylvania State University, State College, PA, United States; 12 Huck Institutes of the Life Sciences, The Pennsylvania State University, State College, PA, United States; 13 Department of Ecology and Evolutionary Biology, Yale University, New Haven, CT, United States; 14 Program in Microbiology, Yale School of Medicine, New Haven, CT, United States; 15 Department of Ecology and Evolutionary Biology, University of California, Los Angeles, Los Angeles, CA, United States

**Keywords:** evolutionary medicine, resistance, adaptation, trade-off, mismatch, life-history, innovation, public health

## Abstract

Evolutionary medicine – i.e. the application of insights from evolution and ecology to biomedicine – has tremendous untapped potential to spark transformational innovation in biomedical research, clinical care and public health. Fundamentally, a systematic mapping across the full diversity of life is required to identify animal model systems for disease vulnerability, resistance, and counter-resistance that could lead to novel clinical treatments. Evolutionary dynamics should guide novel therapeutic approaches that target the development of treatment resistance in cancers (e.g., *via* adaptive or extinction therapy) and antimicrobial resistance (e.g., *via* innovations in chemistry, antimicrobial usage, and phage therapy). With respect to public health, the insight that many modern human pathologies (e.g., obesity) result from mismatches between the ecologies in which we evolved and our modern environments has important implications for disease prevention. Life-history evolution can also shed important light on patterns of disease burden, for example in reproductive health. Experience during the COVID-19 (SARS-CoV-2) pandemic has underlined the critical role of evolutionary dynamics (e.g., with respect to virulence and transmissibility) in predicting and managing this and future pandemics, and in using evolutionary principles to understand and address aspects of human behavior that impede biomedical innovation and public health (e.g., unhealthy behaviors and vaccine hesitancy). In conclusion, greater interdisciplinary collaboration is vital to systematically leverage the insight-generating power of evolutionary medicine to better understand, prevent, and treat existing and emerging threats to human, animal, and planetary health.

## Introduction

Evolutionary medicine (sometimes called Darwinian medicine) applies insights from ecology and evolution to inform, direct, and ultimately improve biomedical research and public health and clinical care. Williams and Nesse’s pioneering 1991 review in *The Quarterly Review of Biology* (1) reimagined vulnerability to disease as the product of evolutionary processes underscoring the importance of historical, functional, and contextual perspectives for the field of medicine (2, 3). While evolutionary medicine does not yet occupy a central position in the curricula of traditional medical education (4, 5), a rapidly expanding, multidisciplinary body of evolutionary medicine scholarship is providing a varied and growing audience of clinicians, researchers, students, and policymakers with a framework for understanding how evolutionary insights can inform the domains of medical education, clinical practice, and public health. What remains significantly less explored is the potential for evolutionary medicine’s diverse approaches to accelerate biomedical innovation.

Here we describe the biomedicine research areas in which evolutionary approaches continue to spark transformational innovation (Box 1) and propose a research agenda to extend this work (Box 2). Evolutionary insights are strengthening the work of cancer biologists, vaccine developers, and many others, and in several fields these insights are already upending orthodoxies, challenging long-standing approaches, and producing exciting, novel therapeutic approaches (6, 7). The novel insights emerging from these evolutionarily informed investigations and innovations illustrate their potential and can help create a roadmap for future work.

Crucially, the systematic study of the full diversity of life exposes the invaluable yet unrecognized model systems that surround us. By focusing on the dynamic interdependence of biological organisms and environments, evolutionary medicine recasts pathology and the body’s countering mechanisms in terms of resistance and counter-resistance. Without an evolutionary framework, these insights – and the potential gains for patients – would go unrealized.

Characterizing the almost inevitable evolution of resistance to medical therapies – whether anticancer drug resistance among cancers or antimicrobial resistance among pathogenic microbes – is one example of how evolutionary perspectives are transforming our understanding of the natural history of disease itself (3). An evolutionary lens is necessary to identify strategies to prevent resistance, and to develop new therapies and algorithms that target the evolutionary process itself. For example, viewing cancer as an ecological and evolutionary problem of controlling ‘cheaters’ (i.e., cancer cells that cheat within the cooperative systems that sustain multicellular life, to the detriment of the organism) offers actionable novel therapeutic insights and adaptive therapies that aim to prevent the evolution of chemotherapeutic resistance. Another example is phage therapy, which provides ways to treat bacterial infections without generating antibiotic resistance and thereby offers a remarkable opportunity to evolution-proof lifesaving treatments. Key challenges include preventing phage resistance.

Evolutionary medicine is not focused simply on improving patient health though better clinical care; it also has the potential to benefit several important areas of public health. The recognition that many modern human pathologies result from mismatches between ancient ecologies, in which much human physiology evolved, and our modern environments has recast health and disease in a broader evolutionary context. Life-history evolution emphasizes the understanding of historically important developmental trajectories and environments to contextualize mismatches that may create pathology. Insights from evolved life-history traits and environmental mismatches have important implications for public health policy.

We must also apply lessons from evolutionary medicine to prepare ourselves for a future life with COVID-19 and other novel zoonotic diseases. Evolutionary models are essential for both predicting and managing pathogenic outbreaks. Also, experience gained during COVID-19 has underscored the fundamental importance of human behavior to the success of medical and public health responses to pandemics. For example, human resistance to the use of novel biomedical innovations – manifested as vaccine hesitancy – has undermined the effectiveness of the extraordinary mRNA vaccines against COVID-19. Thus, to properly apply new ecological and evolutionary insights we must understand these forms of human resistance and how to overcome them. Key to this is recognizing that natural selection works to maximize lifetime reproductive success not survival, and that humans are extremely heterogeneous – meaning that “one-size-fits-all” solutions will not address any substantial problem, including vaccine hesitancy.

The following forward-looking sections explain how ecological and evolutionary approaches have tremendous – and as yet unrealized – potential for improving human health and wellbeing.

## Adaptation as innovation

**FIGURE 1:**
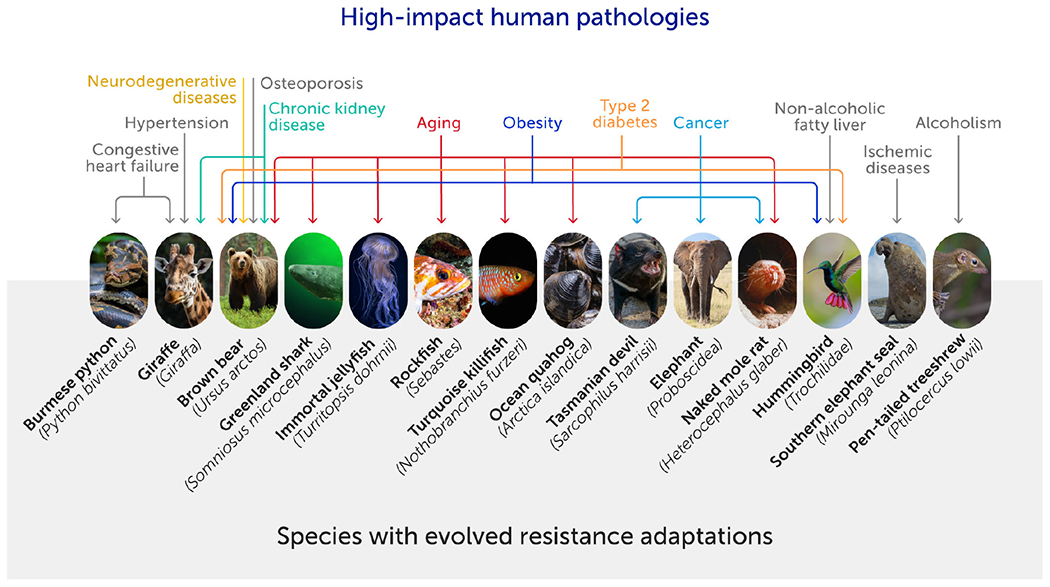
Some animal species have unique physiologies that may confer resistance to a range of high-impact modern human pathologies (8). These evolved physiological adaptations emerge in response to extreme challenges. The identification of species and the unique physiologies that limit vulnerability to disease can accelerate biomedical innovation. A systematic approach is needed to create a comprehensive phylogenetic map of vulnerability and resistance to disease.

Evolved physiological adaptations offer some of the most remarkable examples of biodiversity found in the natural world (8). Contained within the varied and complex physiological systems of other animals may be resistance mechanisms that have important implications for human health (Figure 1). Evolutionary processes have produced many unique physiological adaptations that enhance animals’ abilities to survive and thrive in dramatically varied environments (8). Some adaptations appear to have been driven by natural selection to confer protection against common human pathologies, including infections, cancers, and cardiovascular diseases (8, 9). Given this phylogenetic diversity, countless resistance physiologies are likely to abound in nature, but many areas of human disease have yet to be studied systematically across species.

Only recently has it been possible to identify strategies within the physiological systems of a broad array of other species to counter complex human pathophysiology. Several factors have limited this quest. First, researchers and professionals in human health may have little awareness of the phylogenetically widespread vulnerabilities or resistances to so-called “human” diseases – infectious or noncommunicable – existing in nature (10). Inadequate knowledge exchange between human and veterinary medical fields has fostered an environment in which professionals in human health may lack a fundamental understanding of the range of health challenges shared by both humans and nonhuman animals. For example, the biology underlying pathologies associated with human lifestyles, including atherosclerosis and other cardiovascular syndromes, type 2 diabetes mellitus, melanoma, mammary and lung cancer, cataracts, arthritis, and many other disorders, is shared broadly across species, also placing these animals at risk (11, 12).

The reduction in noncommunicable diseases through improved diet, increased activity, and a decline in disease-promoting practices (such as smoking) has provided strong evidence for modern human culture as a primary “cause” of disease (13). This emphasis has been a cornerstone of preventative medicine and the source of lifestyle modifications reducing the risk to individuals. Yet, the important capacity of human lifestyle changes to modify vulnerability to disease appears to have obscured a parallel, but no less formidable, source of vulnerability to disease: the vulnerability emerging from adaptive evolutionary processes that have shaped the physiological characteristics of every species.

Uncovering differences in species-specific vulnerability is the first step in identifying natural animal models that can provide a blueprint for improving human health. The challenge, however, is that the absence of disease in an individual animal may reflect nothing more than a lack of environmental exposure. Vulnerability is a hidden trait that becomes apparent only when disease emerges, often because of an individual’s unique genetic profile and history of exposure. Despite the challenges in ascertaining the relative disease vulnerability across and between species, the effort is warranted because there may be adaptations conferring enhanced resistance or vulnerability to many diseases that collectively confer much of the global disease burden (14). Findings at either end of the spectrum of vulnerability may lead to novel insights with the potential to accelerate biomedical innovation.

Identifying the biological mechanisms that underlie disease resistances can provide investigators with a blueprint to generate strategies to counter these threats. However, such mechanisms must be identified before they can be studied. Thus, identifying evolved adaptations in other species (i.e. naturally occurring animal models) should be of significant interest to clinicians and investigators treating and studying high-impact human diseases.

Future insights require a systematic approach to identifying these naturally occurring models of resistance (Figure 1). This calls for a comprehensive phylogenetic mapping of vulnerability to specific diseases; with this it will be possible to identify taxa that are particularly resilient or vulnerable using the recognized power of formal phylogenetic comparative analyses (15, 16). Creating these maps is a collective effort that requires the establishment and archiving of standardized necropsy reports from captive and free-living animals (17). Such an expanded comparative database could revolutionize the way in which we identify unique traits and transform these insights into life-saving strategies for the future. Much as recent databases of human genomes are now paying clinical dividends, we expect that expanded necropsy databases could, within a decade, help identify new model systems and unique evolved mechanisms that will focus consequential biomedical research and ultimately lead to novel clinical treatments.

## Targeting the evolution of resistance in cancer

One of the most important frontiers in cancer biology is to develop treatments and cancer-prevention strategies that support the cooperation of the normal cells in our body and help our cells detect and effectively respond to cellular cheating. These treatments and prevention strategies can help support “cheater-detection” systems that operate on every level, including cell intrinsic, neighborhood, and systemic levels. We can use insights from cheater detection in human societies (18) to develop and test hypotheses relevant to treating cancer (19).

Unfortunately, many cancer cells can re-evolve cooperative traits that are then expressed toward other cancer cells, increasing the viability of those aggregations of cancer cells within the body (20). Cancer cells are essentially “pre-adapted” to be able to cooperate with each other because they originated as multicellular cells that have many adaptations for signaling and functioning in coordination with the cells around them (21). Cancer cells often metastasize in groups (22) and these groups exhibit transient cooperation (23), as is expected by evolutionary cooperation theory (24, 25). In addition to supporting the body’s natural cooperation enforcement systems, it is also critical to develop treatments and prevention strategies that undermine cooperation among cancer cells. Some strategies for disrupting cancer cell cooperation include interfering with the adhesion factors or signaling factors used by these cancer cell aggregations (20, 26).

Cancer is a disease of somatic evolution, meaning that it happens because cells inside the body evolve in ways that further their own fitness interests at the expense of the body. But somatic evolution is not always harmful. Elucidating how somatic evolution can contribute to our resistance to disease is an exciting area for new work. For example, the immune system uses somatic evolution to create variants of antibodies, allowing us to respond more effectively to rapidly evolving infections (27). This is an example of the body harnessing the power of somatic evolution to fight evolving entities whose interests conflict with our own. Also, there may be some benign neoplasms that can “crowd out” malignant neoplasms by taking up ecological space, and the identification of the mutations underlying these benign neoplasms could offer new therapeutic strategies.

Treating cancer using an evolutionarily informed perspective is also about considering the evolutionary consequences of treatment for the trajectory of the cancer before and during treatment. Several approaches, including adaptive therapy and extinction therapy, are informed by the evolutionary and ecological dynamics underlying tumor responses to treatment. Both approaches reject the idea of using an anticancer drug at a high dose for as long as it works, because extended exposure to high doses selects for cells resistant to the drug used. Adaptive therapy aims to stabilize the tumor size and maintain a population of drug-sensitive cells, allowing the drug to be used for long-term tumor control (28). Adaptive therapy has been successful in treating advanced prostate cancer in a clinical trial (29) and it remains one of the most promising strategies for long-term control of advanced cancers, though more clinical trials are needed across a broader range of cancers. In extinction therapy, one drug used initially to reduce the cancer cell population size is stopped (while it is still working) and replaced by a second drug. This is based on principles derived from extinction biology where a decrease in population size, followed by new ecological perturbations, is often associated with population extinction (30, 31). This approach is in the very early stages in terms of being tested and used in the clinic.

Because adaptive therapy and extinction therapy leverage the ability to respond to changing information about the status of the tumor during therapy, they are highly general approaches that hold the greatest promise as “universal” principles for developing new cancer therapies. It is widely recognized that the evolution of resistance poses a critical clinical problem because drug-resistant cancers can no longer be treated, leading to progression (29, 32–34), though there is a need for epidemiological studies to quantify the extent of this problem. Drug toxicity is another problem that affects both mortality and quality of life among cancer patients. Evolutionarily informed approaches, such as adaptive therapy and extinction therapy, could solve this problem because they anticipate the evolutionary dynamics and shape the evolution of the tumor in ways that allow for long-term cancer control and significantly reduce the population size of the tumor to the point where it may be possible to essentially cure it with much lower doses, according to models (30).

We are susceptible to cancer because we are multicellular organisms, but we are also highly resistant to cancer because we have undergone millions of years of evolution as cooperative societies of cells that effectively detect and respond to cellular cheating. All multicellular organisms are societies of cells operating in precarious cooperation, and an evolutionary and ecological approach can help mitigate such precariousness. This approach can also help to rebalance the evolutionary and ecological dynamics in our bodies when they are compromised by cancer cells, using approaches such as adaptive therapy and extinction therapy to leverage evolution and ecology to reduce the burden of cancer and, in some cases, even drive cancer to extinction within the body.

## Targeting the evolution of antimicrobial resistance

The effectiveness with which antimicrobial drugs, insecticides, and anticancer drugs kill their targets is also their undoing. By killing drug-sensitive life, they create the conditions in which natural selection drives the evolution of drug and insecticide resistance (35). To simplify the discussion on how evolutionary-inspired insights can help solve this problem, we focus primarily on the resistance problem in the context of antimicrobials (antivirals, antibiotics, antiparasitics).

The conventional solution to the problem of antimicrobial resistance (AMR) is to find new chemical agents to replace those that fail. The sustainability of this drug-discovery treadmill is not obvious, especially given that resistance mechanisms are getting ever more generic (some bacteria have evolved efflux pumps that can expel drugs not yet invented), and the costs of bringing new products to market can cripple the process of translating laboratory science into approved drugs (36, 37). On economic grounds alone (38, 39), there is an overwhelming case for shifting the focus from developing replacement products that will eventually fail to tackling the central problem: the evolutionary process itself.

Applying evolutionary insights to antimicrobial drug development has the potential to spark transformational innovation that saves lives. Of these, the cheapest are innovations that make better use of existing drugs. A key historical example was the discovery of evolution-proof treatment regimens for HIV and tuberculosis (40–43). There, the right combination of antivirals or antibiotics can raise the genetic barrier to resistance so high that all mutants remain susceptible to at least one partner drug. Consequently, resistance to the combination never arises. These anti-evolution regimens continue to deliver massive health gains globally. Other ways of using existing drugs may also offer important gains. For example, using alternate drugs over time (cycling) or space (mixing) at a patient, hospital, or community/national level has the potential to improve treatment. While the levels of evidence supporting this approach are mixed, the integration of resistance data into evolutionary model development may produce more compelling results in the future (44–46).

Similar theory-data interplay should determine the optimal solution to a quandary at the heart of much current practice in antimicrobial stewardship. Conventional wisdom posits that when treating patients, it is important to use drugs to rapidly eliminate cells that might mutate into resistant variants. But this conventional approach can be counterproductive in that it increases the evolutionary advantage of any resistant mutants that can survive this treatment. In almost all cases, the optimal treatment regimen (dose, duration, interdose interval) that maximizes patient health while minimizing AMR evolution has yet to be determined empirically. However, several clinical trials have revealed that treatments often continue for too long (47), while animal studies often show that conventional dosing regimens are too low or too high (48, 49). As with the promise of lower doses and evolutionarily based algorithms in cancer treatment (i.e. adaptive therapy, discussed above), there is a need for new dosing and drug scheduling regimes that are responsive to the evolutionary processes that underlie infectious diseases and AMR (50, 51). Indeed, there may be situations where a patient will live longer if no attempt is made to cure the person with antimicrobial chemotherapy, but instead drugs are used to contain symptoms and spread. Evolutionary thinking provides a framework in which to consider that agonizing clinical decision (52).

A completely different approach to the resistance problem is to develop chemistries that target the evolutionary process itself. It is tempting to call these chemistries “anti-evolution drugs,” although they are not drugs in the conventional sense. Their primary aim is not to kill the target organisms (though they might do that), but rather to inhibit resistance evolution. The speed of AMR evolution, like all adaptation, depends on the genetic variation present and the strength of selection. Compounds can be identified that can reduce both genetic variation and the strength of selection.

Horizontal transfer of genes from resistant bacteria to sensitive bacteria is a key means by which antibiotic resistance can spread quickly. Drugs are being developed that interfere with genetic transfer mechanisms, such as conjugation (53). Another approach would be to use drugs that kill only those bacteria with specific resistance mechanisms. Used as adjuvant therapy, these would protect a conventional antibiotic from resistance development because any rare mutant with that resistance mechanism would be selectively eliminated immediately. Resistance to the adjuvant would not arise because the rare mutant would be killed before it could acquire resistance to the adjuvant, and the adjuvant does not select for resistance in the remaining bacteria because it does them no harm.

**FIGURE 2:**
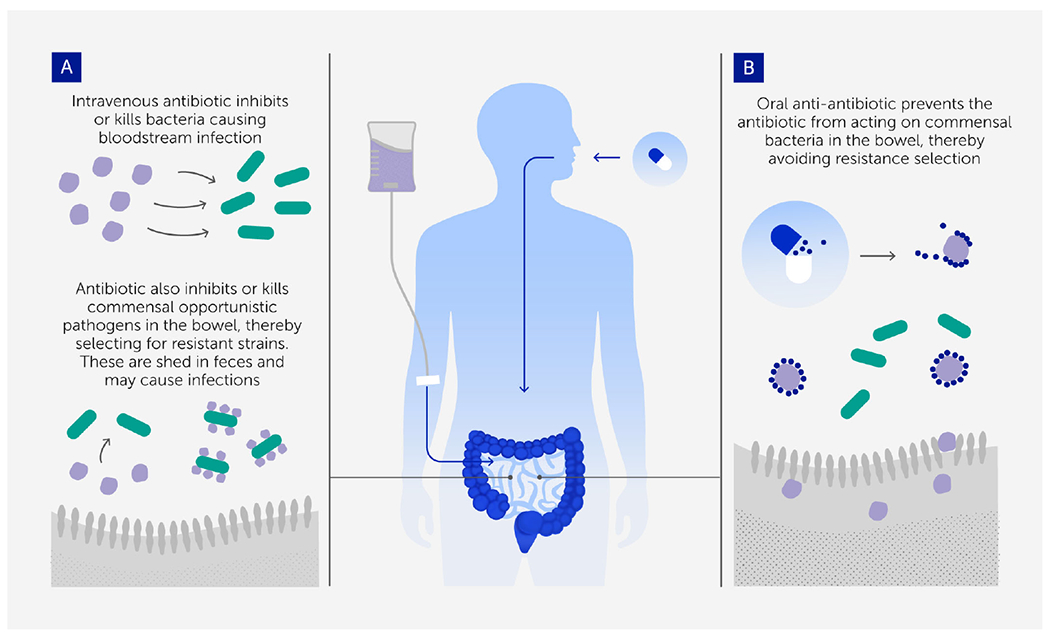
The anti-antibiotic strategy for preventing resistance evolution and onward transmission in colonizing opportunistic pathogens (54). **(A)** Intravenous (IV) antibiotics are needed to treat bloodstream infections, but a small proportion passes to the gastrointestinal tract and selects for resistance in colonizing bacterial populations that are the source of the onward transmission of many hospital-acquired infections. **(B)** Oral adjuvant therapy with a compound that inactivates antibiotics in the gastrointestinal tract (anti-antibiotic) removes that selection so that the transmitted populations are no longer resistant.

Drugs can also be used to reduce the strength of selection for resistance. Anti-antibiotics are one approach (Figure 2). These compounds inactivate any intravenous antibiotics that reach the gastrointestinal tract to prevent resistance in off-target bacteria. This can maintain the effectiveness of the antibiotics in the rest of the body while eliminating the selective pressure favoring the evolution of drug resistance among microbes in the gut, thus preventing resistance evolution in many hospital-acquired infections (55, 56). Indeed, inactivation of an antibiotic such as vancomycin in the gastrointestinal tract might tip the balance in favor of vancomycin-sensitive bacteria, thus reversing decades of resistance evolution. Both laboratory and clinical data show that anti-antibiotics can help mitigate antibiotic resistance and, in some cases, can even completely prevent it (56). Likely there are many anti-antibiotic chemistries yet to be discovered beyond the existing anion exchange resins (56), beta-lactamases (57) and activated charcoal (58).

Chemistries that inactivate host-derived compounds could also provide innovative resistance management. AMR selection depends on the relative competitive advantage of resistant and sensitive pathogens in drug-treated hosts. Resistant mutants have an advantage in drug-treated hosts because the drug reduces the competitive suppression that drug-sensitive progenitors would otherwise exert (59–61). Limiting key resources so as to strengthen competition between drug-sensitive and -resistant pathogens during antimicrobial treatment has been experimentally shown to tip the balance in favor of drug-sensitive microbes, allowing drugs to be curative even in the presence of resistant mutants (62, 63). This raises the prospect of adjuvant drugs that could transiently inactivate or remove nutrients that drive the competition between resistant and sensitive microbes – thereby allowing drug treatment without resistance emergence. Similar evolution-proofing could be achieved with treatment regimens aimed at reducing pathogen burdens to alleviate symptoms while maintaining sufficient sensitivities to competitively suppress resistance (64), analogous to adaptive therapy in cancer (50).

## Phage therapy

In 2017, the World Health Organization (WHO) highlighted the threat of Gram-negative bacterial pathogens resistant to multiple antibiotics (65). The discovery, design, and development of new and alternative antibacterial therapies are deemed crucial because antibiotics are failing widely. “Phage therapy” uses lytic bacteriophages (or simply, phages: viruses that specifically infect and kill bacteria) as bactericidal agents to combat infections. This approach was first developed in the early 1900s (66, 67), predating Alexander Fleming’s accidental discovery of antibiotics in 1928. Until recently, Western medicine has largely ignored phage therapy as a possible alternative to conventional antibiotics, whereas this method has been used for decades in countries such as the USSR (and now, Russia), Poland, and Georgia – where the George Eliava Institute of Bacteriophages, Microbiology and Virology has operated since the 1930s (68, 69).

During its infection cycle, a lytic phage will: attach to receptor (s) on the cell surface of bacteria and deliver its DNA or RNA to the cytoplasm; undergo viral replication in the cytosol *via* bacterial transcription, translation, and replication; and upon formation of new phage particles, escape the cytoplasm through lysis (death) of the host bacterium. This process is then repeated by the new phage particles as they infect additional susceptible cells. These steps highlight a long-understood benefit of phage therapy: utilizing lytic viruses as self-amplifying “drugs” that target and kill susceptible cells may be more efficient than applying antibiotics that are incapable of self-amplification. Although other intracellular replication strategies have evolved in phages, these obligately lytic (or “virulent”) phages are most often developed for use in phage therapy.

**FIGURE 3:**
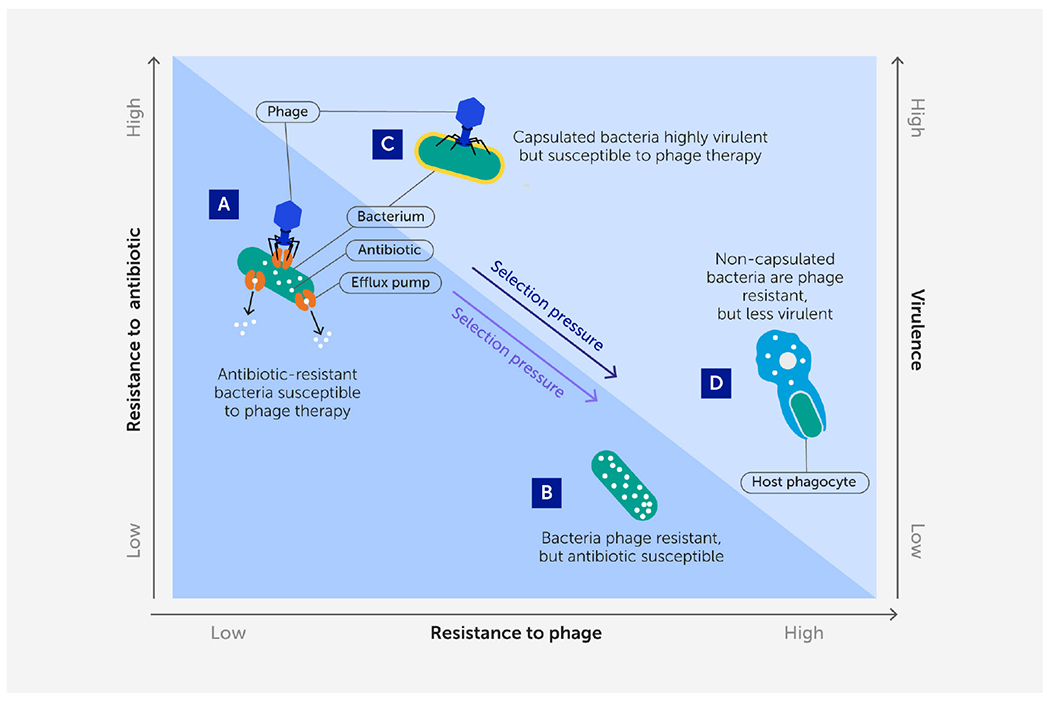
A renewed approach to phage therapy: phage selection against antibiotic resistance or virulence. Certain lytic phages may be more effective in phage therapy because they kill target bacteria while simultaneously imposing strong selection against bacterial antibiotic resistance or virulence when bacteria mutate to avoid phage attack. **(A, B)** Phages that use antibiotic efflux pumps as receptors **(A)** can select for phage-resistant bacterial mutants with impaired efflux pumps **(B)** that are more sensitive to antibiotics (72). **(C, D)** Phages that bind to structural virulence factors such as a capsular antigen **(C)** can select for phage-resistant non-capsulated bacterial mutants **(D)** (73) that are less virulent because they are more easily engulfed by host phagocytes (74).

However, one obvious limitation to phage therapy is the inevitable evolution of phage resistance in the target bacteria (70, 71). Modern approaches to phage therapy should both acknowledge and capitalize on this certainty (Figure 3). Evolutionary biology describes how genetic trade-offs should be widely observed in biological systems; organisms sometimes evolve one trait that improves fitness (a relative advantage in survival or reproduction), while simultaneously suffering reduced performance in another trait (75–77). These trade-offs were observed in classic phage-therapy studies in mice (73), which showed that the evolution of phage resistance in bacteria can impose costs for other bacterial traits that are therapeutically beneficial, such as decreased virulence, antibiotic resensitization, and host-colonization deficiencies in pathogenic bacteria.

Phage therapy would thus benefit from an evolutionary medicine approach; utilizing a certain phage should select for the target bacterial pathogen to evolve phage resistance, while concomitantly suffering a specific genetic trade-off that would be biomedically useful (72, 78, 79). Which phages should be expected to select for these beneficial trade-offs? If the proximate binding of a lytic phage is known to associate with a virulence factor or mechanism for antibiotic resistance in the target bacteria, this should exert strong selection for the bacteria to mutate or down-regulate the phage-binding target(s). This approach should be especially useful in the case of opportunistic bacterial pathogens because the bacteria could evolve reduced virulence or antibiotic resistance and still thrive in a different ecological setting (e.g., soil), as opposed to the “arms-race” selection for escalating virulence in an obligate pathogen – such as in response to vaccine pressure (e.g., in the Marek’s disease virus in chickens) (80). Thus, this approach to phage therapy should be doubly effective: success is achieved when phage lyse the target bacterium, but also when bacteria evolve phage resistance because they suffer reduced virulence or increased sensitivity to antibiotics (Figure 3).

Recent studies demonstrate that selection for evolved phage resistance can cause the target bacteria to become less pathogenic, owing to a trade-off involving decreased virulence. Selection imposed by various enterococcus-specific phages led to phage-resistance mutations in the enterococcal polysaccharide antigen (*epa*) gene cluster of *E. faecalis* bacteria, where the mutants harbored altered cell-surface properties and were deficient in intestinal colonization and transmission in mice (81). Likewise, structures used in bacterial motility (e.g., pili and flagella) can serve as binding targets for phages, causing bacteria to evolve phage resistance by eliminating motility, which can reduce bacterial virulence (82, 83). A last example is the ability of phages to bind to OmpA membrane proteins of *Shigella flexneri* diarrheagenic bacteria, which exerts selection for phage resistance *via* change or elimination of this protein, preventing intercellular movement of the pathogen between human intestinal cells (84).

Similarly, evolution of phage resistance can cause target bacteria to become resensitized to chemical antibiotics, which would beneficially extend the usefulness of drugs in our waning antibiotic arsenal. For example, a phage that attaches to an antibiotic efflux pump to initiate infection may select against the expression of the efflux pump responsible for actively removing drugs from the cell, rendering the bacteria more sensitive to antibiotics that were previously effluxed (72). Yet, a few phage-resistant mutations potentiated antibiotic resistance in *E. coli in vitro* (i.e., a trade-up, not a trade-off, in bacterial traits) (85). This demonstrated that pleiotropic interactions are possible for bacterial traits and the molecular mechanisms underlying evolutionary trade-offs should be investigated closely when developing phages for therapy (84, 86).

Notable successes in emergency phage treatment of antibiotic-resistant infections (87–89) bolster confidence that phage therapeutics could be widely adopted, pending supportive outcomes in ongoing clinical trials (90). The evolutionary-medicine approach of using phage selection to “steer” target bacteria to evolve phage resistance by trading pathogenicity has enabled more effective selection of phages in emergency patient treatment. Favorable outcomes using these approaches (88) illustrate the value of evolutionary considerations in shaping the rational choice of phages for treatments that minimize (or even capitalize on) the inevitable evolution of phage resistance in target bacterial pathogens. Deeply informed by evolutionary thinking, phage therapies are helping to create strategies of the future that can effectively counter both pathogens and antimicrobial resistance.

## Life-history evolution

**FIGURE 4:**
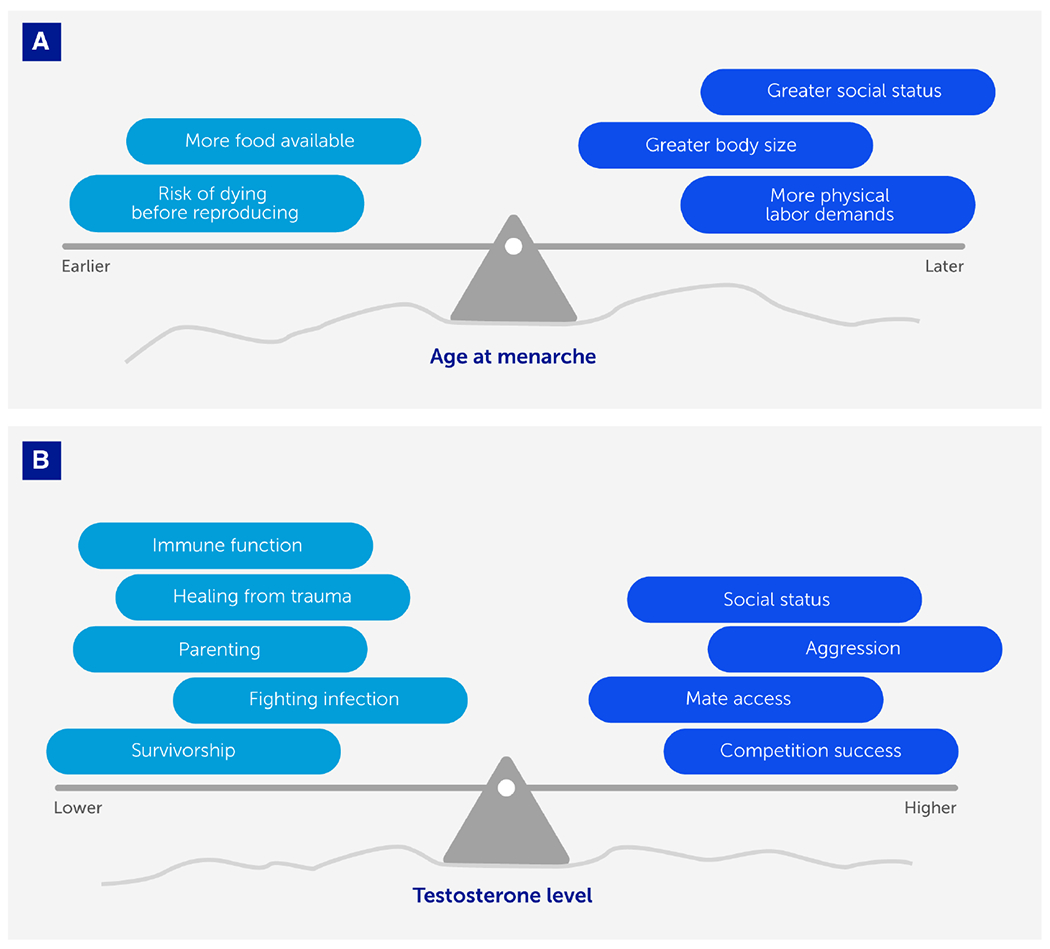
Life-history trade-offs optimize fitness (with respect to constraints) in response to ecological and social contexts. We depict two examples showing how life-history traits (age at menarche and testosterone levels) vary between individuals in ways that reflect adaptive strategies optimized for different ecological and social conditions (represented here by the uneven surface, or landscape). As those conditions change, the trade-offs (represented by weights that tip the balance of the see-saw or teeter-totter) governing life-history traits change concomitantly. **(A)** In females, it is adaptive for age at menarche to occur earlier or later depending on various conditions, with fitness advantages of earlier menarche (e.g., minimizing risk of dying before reproducing) weighed against fitness advantages of later menarche (e.g., greater embodied resources to support a healthier pregnancy) depending on the ecological and social context. **(B)** In males, it is adaptive for testosterone levels to be higher when the fitness landscape favors engaging in competition with conspecifics and gaining social status, and lower when the fitness landscape favors investing in parenting effort or healing.

Advances in evolutionary medicine are not restricted to studying the evolution of disease vulnerability and resistance; other advances will emerge from recognizing the importance of mismatches between evolved life-history traits and the current environment. Life-history traits include growth, reproduction, and survivorship dynamics (91, 92). Time and energy are allocated competitively toward each of these domains (75). Selection optimizes the balance between these investments in ways that are sensitive to ecological and demographic conditions. Life-history perspectives can identify epidemiological trends in the future and past, clarify when inter-individual differences are pathological or adaptive, and clarify trade-offs that explain – for example – why fighting infection is not always an adaptive priority (Figure 4).

Women’s reproductive life-history norms have changed profoundly in recent history compared to patterns that would have predominated throughout our species’ evolutionary past (93). In the future, we anticipate continued, large-scale changes in reproductive norms such as the precipitously falling number of offspring per woman (94). Thus, understanding the relationships between female reproductive life-history traits and disease risks has clear public health applications for forecasting future disease burdens (95). An important example is women’s reproductive life-history, as this affects a woman’s acute, short-term and long-term health. As an example of a life-history event, pregnancy may alter a woman’s risk of hypertension during the event (96), the risk of depression in the short term postpartum (97), and the risk of pelvic organ prolapse later in life (98). Therefore, changes in our species’ norms of age at first birth, number of pregnancies, and inter-pregnancy intervals have implications for changes in health and disease, and hence for public health systems.

We surmise premodern life-history norms based on comparisons with contemporary small-scale societies, and some information can be gleaned from the fossil record. Both methods give us only a flawed and limited estimate of our past (99, 100). Nonetheless, important differences emerge, such as older age at menarche, younger age at first birth, longer duration of breastfeeding per child, and greater completed fertility per woman, compared with contemporaneous post-industrial peoples (101). The disparities in life-history patterns suggest that diseases for which risk or resilience is tied to life-history events are very likely different for most people today compared with our premodern counterparts (102).

Breastfeeding represents a domain in which, arguably, the largest change in life-history norms has occurred across history. Breastfeeding has been correlated with lower risk of type 2 diabetes, hypertension, cardiovascular disease, metabolic syndrome, myocardial infarction, breast cancer, and Alzheimer’s disease in mothers (103). Therefore, we surmise that breastfeeding-attributed resilience was greater in previous eras, and concomitant risks will continue to change as breastfeeding rates change in the future.

Over the past century, girls have been exhibiting progressively earlier age at reproductive maturation across most parts of the globe (104). A biomedical perspective pathologizes this pattern as precocious puberty, in search of toxic culprits (105). However, life-history theory elucidates how conditions encountered during early life calibrate sexual debut to occur on a flexible timeline that optimizes fitness (106). It is adaptive for girls to strategically aim to have their first pregnancy when their bodies and circumstances can support a healthy pregnancy and infant (107). Across history, as physical labor demands diminished and food availability improved, constraints on juvenile growth were relatively relieved, so girls garnered sufficient embodied resources to begin their reproductive life phase at a relatively earlier age.

While especially good conditions can promote earlier puberty, so can especially bad conditions. Harsh environments encountered during early life have been associated with earlier reproductive maturation for girls (108, 109). While a biomedical perspective casts this phenomenon as impaired health caused by deprivation or trauma, an evolutionary perspective suggests that these life-history strategies are optimally suited for an individual’s anticipated ecological context (110, 111). It is adaptive in low-risk conditions to delay puberty to achieve greater body size and social status, and in high-risk conditions to accelerate puberty to avoid risk of death before reproducing (92, 110).

Life-history trade-offs sometimes compromise disease defenses in favor of reproductive effort. An evolutionary perspective highlights the role of testosterone in modulating life-history allocations in ways that maximize fitness in various conditions (112). The biomedical perspective supposes that low testosterone reflects pathology (113) and there is enormous interest in improving men’s testosterone levels, ostensibly by increasing them (114, 115). In contrast, an evolutionary perspective highlights how testosterone modulates the trade-off between investment in survival versus reproduction, and that variations in testosterone levels can be adaptive in different contexts (112, 116). Across many species, males’ testosterone is elevated during intrasexual competition, presumably to gain social status that garners access to a mate (117, 118). Elevated testosterone comes at the cost of immunosuppression (although the details are debated) and behaviors (e.g., aggression) that undermine pair bonding and paternal behavior (112, 119). In response to illness or trauma, testosterone levels may diminish as a facultatively adaptive strategy to promote immune function and discourage risky behaviors, i.e. prioritizing investment in survivorship over reproduction (118, 120). Future biomedical research could benefit by applying an evolutionary understanding of the role of testosterone to help improve the mental and physical health of men.

Evolutionary perspectives informed by life-history theory (Figure 4) have been an important source of insights regarding disease risk, etiology, and treatment. However, there is still tremendous untapped potential for utilizing this approach to better understand trajectories of change in physiology (including hormone levels and immune function) over the course of the lifespan and whether these are part of the “normal” human lifetime trajectory, which involves adaptive trade-offs and responses to environmental and social cues. In the future, life-history perspectives will help recast pathologies as part of broader normative responses shaped by the environment in which an organism develops and ages.

It is not feasible or efficient to provide all disease prevention interventions to all people. Therefore, it is crucial to identify at-risk groups to whom clinicians can offer targeted, appropriate interventions as early as possible. A better understanding of how life-history traits relate to disease etiology and risk can help us determine whether specific individuals at high risk of certain diseases can be identified based on certain life-history patterns. Using a short life-history interview or medical chart review to identify these individuals would be clinically easy, cheaper, and simpler than screening methods that involve biomarkers or neuroimaging. The ability to identify high-risk individuals based on certain life-history patterns would open up new opportunities to intervene preemptively.

## Evolutionary medicine and public health

As niche modifiers that continually change their environment regardless of adaptive need or pressures, humans do not simply sustain a constant niche (121). Instead, their cumulative technological and environmental actions can affect the environment in ways that exceed their physiological adaptive capacities, leading to *evolutionary mismatch*. Evolutionary mismatch occurs when an organism is exposed to an environment that exceeds its adaptive capacities, and it is one of the core pathways by which evolutionary processes can lead to ill health (122). It can be exacerbated by environmental signals acting in early development, reflecting the related concept of *developmental mismatch* – where an organism develops its physiology according to present cues but later encounters an environment mismatched to its physiological phenotype (123). Public health is concerned with broad population-based trends in morbidity and ways to reduce the impacts; in this regard, both concepts of mismatch are growing in importance in the consideration of public health policies.

The clearest example is obesity and its associated morbidities of type 2 diabetes and cardiovascular disease. In high-income societies, rates of noncommunicable disease have risen rapidly since World War 2 and are generally associated with the rapid development, industrialization, and marketing of processed foods. Traditionally these diseases have been treated as “lifestyle” diseases, with interventions focused on the individual, such as exercise and dietary changes (124, 125). Yet, there is also compelling evidence that once obesity is established, it creates new physiological set points that make it difficult to reverse it permanently (126). There are now sufficient ecological incentives operating to shift the focus from the individual to the population; this recognition is gradually entering public policy; for example, *via* food taxes and restrictions on food marketing to children.

The evolutionary mismatch presented by the modern nutritional ecosystem may be further compounded by developmental factors. For example, fetal undernutrition can induce adaptive regulatory responses that are appropriate for postnatal low-nutrition environments but not for high-nutrition environments (123). The resulting developmental mismatch increases the risk of disease, thus creating a public health imperative to improve nutrition before and during pregnancy. On the other hand, maternal obesity before and during pregnancy – especially if associated with gestational diabetes – can also induce fetal responses that lead to a greater risk of adiposity and diabetes in offspring (127). Unlike other aspects of placental physiology, the placenta does not limit glucose transfer to the fetus, resulting in fetal hyperinsulinemia with adipogenic consequences. This pathway to ill health involves *evolutionary novelty* and *mismatch*; that is, exposure to an environment not previously encountered in the individual’s evolutionary history (e.g., the consequences of maternal obesity). Similarly, the replacement of breast milk with artificial formula is an evolutionary novelty with long-term metabolic outcomes (128).

In this context, labeling these cardiometabolic morbidities as lifestyle diseases puts the focus – and onus for interventions – on the individual. It has been suggested that a more appropriate term might be “Anthropocene-related disease” (ARD), which encompasses the impacts of the modern socioeconomic and physical environment on the health of the population, thus shifting the focus of intervention to population- and systems-based measures (13).

The concept of Anthropocene-related disease can be extended from the evolutionary principles of mismatch. For example, explanations for the rapid rise in the prevalence of allergic disease have generally been based on the “hygiene hypothesis” and the consequences of avoidance of parasites and allergens in early life (129). Other modern interventions such as Cesarean sections, the use of antibiotics, and cow’s milk-based formula feeding from birth may lead to long-term changes in the microbiota with broad health implications (128, 130).

The impact of maternal stress in pregnancy on fetal brain development is well demonstrated in rodents and lagomorphs. For example, maternal stress in the Arctic snowshoe hare (*Lepus americanus*) due to predator exposure has been shown to have adaptive value *via* heightened offspring sensitivity to stressors (131). Recent human studies have similarly linked moderate maternal stress to impaired development of executive functions in children (132). This is hypothesized to reflect how a likely conserved adaptive mechanism of developmental plasticity has now become maladaptive in the modern high-stress social environment, through increased emotional and behavioral dysregulation and attention-span problems interfering with schooling.

A further manifestation of Anthropocene-related disease can be observed in the rapid rise in global prevalence of poor mental health in adolescents (133, 134). The rate of this rise cannot be attributed to individual pathology. Instead, the modern ecological context, which is itself the result of cultural evolution, is likely an underlying reason (13). This new ecological context includes changing social systems resulting in major shifts in child-rearing patterns (e.g., parents insulating children from many risks) and changed personal and societal expectations of young people.

Another emerging challenge for mental health comes from the rise of digital technologies. The emergence of the internet, social media, the metaverse, and other technologies has exposed humans to a fourth “virtual” dimension unlike anything previously experienced by our species (135). This may have major impacts not only on our mental health and psychological resilience, but also on the operation of the social institutions that humans evolved with as social animals. Arguably, the scope of human interaction has become much broader, but also shallower, than that with which we evolved (136). As the pace of technological change is unlikely to slow, the nurturing of those skills most critical to thriving in the digital age (such as psychological resilience, self-control, and empathy) will demand greater focus by education systems and preventative public health.

## COVID-19 and evolution

The centrality of evolutionary medicine to global health was highlighted by the SARS-CoV-2 pandemic that swept around the globe in early 2020. The emergence of a novel zoonotic pathogen into human populations is an evolutionary process in which an often poorly adapted pathogen enters the human population from an animal reservoir. Human-to-human transmission may initially be unlikely, but once it occurs, longer and denser transmission chains then provide the pathogen with more opportunities and time to adapt to the new host and evolve to transmit efficiently. Evolutionary models illustrate the dynamics of this process and highlight the pathogens with the greatest risk of making a zoonotic leap (137–139).

To understand and potentially predict the spread and public health impact of a pandemic, epidemiologists must characterize a virus’s transmissibility, virulence, and potential to reinfect. Even with good estimates of these, forecasting is extremely difficult (140). Moreover, these dimensions are not fixed; they shift as human behavior responds to life during a pandemic, and as the virus evolves. Still, evolutionary theory makes valuable predictions about future scenarios (141) and about which variants are likely to be evolutionarily successful; for example, those that increase transmission early in the pandemic. With COVID-19, we saw immediate evolution and spread of the D614G mutation that increased transmissibility, followed by successive waves of the Alpha, Delta, and Omicron variants. Evolutionary models correctly predicted the properties of the successful variants: transmission advantage is more important than immune escape, particularly early in a pandemic, and variants capable of reinfecting are successful only when also exhibiting high transmissibility (142).

While of vital importance, the path of virulence evolution is more difficult to predict. There is a popular belief that a virulence-transmissibility trade-off will inevitably lead pathogens to evolve reduced virulence. The basic idea is straightforward: pathogens that keep their hosts alive and mobile will be better able to transmit than those that kill or incapacitate their hosts. Unfortunately, this view has very limited theoretical or empirical support. Even the canonical example, myxoma virus in Australian rabbits, fails to validate this hypothesis. Although this virus initially evolved reduced virulence, it later reversed course and evolved extremely high virulence (143).

Virulence-transmissibility trade-off models assume that virulence changes have appreciable effects on viral fitness, but this is often untrue (144). With COVID-19, for example, severe illness and death typically occur a week or more after the end of the usual infectious period; the fate of the host at that stage has little if any selective impact for the virus, which has likely already transmitted. Even with the trade-off framework, we cannot count on virulence to decline in the early years of an emerging zoonosis. When a pathogen first enters the human population, it is unlikely to be at or near the virulence-transmissibility frontier that is critical to trade-off models. Rather than optimizing along this frontier, initial evolutionary changes simply move toward it – and may involve increases or decreases in virulence, depending on the properties of the initial strain and the happenstance of mutation supply (145). Levin and Bull’s (146) “short-sighted evolution” model of virulence provides another reason why virulence evolution may not lead to predictable declines. With diseases such as polio, virulence is not evolutionarily optimized but rather the result of myopic within-host evolution. The pathogen invades regions such as the central nervous system, but these are evolutionary “dead ends” from which further transmission does not occur. For these reasons, it is not a foregone conclusion that SARS-CoV-2 will evolve to reduced virulence.

The examples thus far draw upon Darwin’s insight about adaptation by natural selection. His other great insight was the branching pattern of common ancestry among all living things, and this insight has also been invaluable for understanding the dynamics of an emerging infection. Genomic regions associated with both severe infection (147) and those that offer some protection against severe infection have been identified in humans that date back to genes inherited from our Neanderthal ancestors (148). Widespread whole-genome sequencing provided an enormous trove of data, with a million SARS-CoV-2 sequences deposited by April 2021 (149). Attwood et al. (150) recently reviewed the use of phylogenetic and phylodynamic techniques during COVID-19 in detail and here we mention a few examples therein. Phylogenetic analysis of COVID-19 sequences obtained in late February 2020 established that SARS-CoV-2 had been circulating undetected for weeks in Washington State (151) and marked a turning point in the US control strategy (152). Phylogenetic analysis also proved valuable in establishing or excluding suspected sites of transmission (153) and helped determine the distribution of secondary cases and the role of superspreading (154). Phylogenetic approaches also provided clues about the processes by which variants of concern emerge: the Alpha and Omicron variants diverge with unusually long branch lengths from other strains (155, 156). This hints that each may have differentiated during a long-term infection, possibly in an immunocompromised individual (157, 158). Indeed, phylogenetic inference and evolutionary theory together suggest that immunocompromised patients may represent an important source of novel variants of concern (159).

Multiple factors limit robust predictions of how the pandemic may progress. For example, therapeutic advances are likely to increase the immunocompromised population. Varying degrees of immunity throughout the global population conferred by vaccines of varying efficacy with different targets may give rise to adaptive opportunities for the virus. Further, numerous SARS-CoV-2-susceptible species have been identified, and anthropozoonotic transmission of COVID-19 has now been documented in species such as the dog, cat, and North American white-tailed deer (160, 161). This poses a risk of human reinfection with variants that have undergone further evolution in another host species. Indeed, zoonotic reinfection of humans has already occurred in mink farms (162).

As the SARS-CoV-2 pandemic continues, evolutionary perspectives remain critical not only to public health management but also to public policy. To reduce future risks of zoonotic disease, international agreements, including the International Health Regulations and a putative pandemic treaty, need to fully reflect the understandings that an evolutionary perspective provides (163).

## Overcoming human resistance to public health measures

One of the most puzzling aspects of human health and behavior is our willingness to knowingly engage in activities that harm our own health. We know that overeating, smoking, taking drugs, driving fast, and fighting are bad for us, and that exercise is good for us. It is clear that vaccines against COVID-19 are extremely effective and safe, so having the vaccine will protect our health. Yet a significant proportion of humanity, including those who are well educated, choose to ignore this advice.

While governments are charged with promoting the public good, individuals are evolved to be concerned about individual Darwinian fitness and the fitness of close relatives (especially those in the younger generations) (164). One of the most important insights from evolutionary medicine is that natural selection operates to maximize inclusive fitness, not simply survival. Life-history theory makes clear there can be trade-offs between traits that help reproduction over those that favor a long life (indeed, reproduction itself entails risks of maternal mortality and infection). Those who are male, poor, at high risk of not finding a mate, at risk of not being able to meet their basic needs, and especially those with children whose immediate needs must be met, are among those for whom risk-taking in the short term is likely to be more important than long-term health considerations (165). These groups encompass much of the population and may include many who were less likely to adhere to social distancing guidelines during the COVID-19 pandemic (166).

Many public health measures are classic public goods dilemmas that can be difficult to enforce. These measures help society but provide only a small or negligible benefit to the individual or their children (such as having a vaccine against a disease that primarily harms older people when you are a young person). Cooperation between unrelated individuals is hard to maintain. Enforcing public health measures through legislation and policing is often impracticable and costly, and can infringe on human rights. A behavioral ecological approach predicts that individual costs and benefits drive behavioral decision-making (167), so manipulating payoffs may be the most effective strategy to drive health behavior change. Many governments have tried to increase voluntary vaccine uptake by incentivizing vaccine adoption *via* cash rewards and lotteries (positive incentives), as well as by increasing the costs of non-compliance, by restricting travel or entrance into social venues for the unvaccinated (negative incentives). Negative incentives helped stimulate an immediate surge in the uptake of COVID-19 vaccines in France (168).

Reputational costs are one of the few ways to enforce cooperation between unrelated individuals (169). However, reputational costs are often very local to one’s social network, so will not be effective if one’s community does not care about or even promotes the harmful behaviors concerned. Some people may adopt bizarre health beliefs (such as outlandish conspiracy theories about vaccines) not because of scientific misunderstandings, but because the belief becomes a social marker of group identity, and the benefits of being part of a cultural or political group are their main priority. Anti-vaccine sentiments can co-evolve with pandemics, flourishing in these small groups – especially when disease risks are low – and helping the disease to re-emerge later (170, 171). Preventing people from joining such groups, or persuading people that such groups have a toxic reputation, may reduce their influence more than scientific arguments about the effectiveness and safety of vaccines.

Some decisions often described as “problematic” are actually not so, and we should respect the heterogeneity in payoffs experienced by different groups within society. For example, if a young woman decides to have a baby when she is young and single, the variety of costs and benefits to her may reflect such factors as the ability of her mother to help her with childcare before the mother succumbs to any age-related health problems (172), and not what education the young woman is missing. Young men who choose not to use condoms may be unconsciously prioritizing reproduction over the risk of HIV infection, especially in environments where there are other, larger mortality risks (173). Costs and benefits to individuals and their children cannot be considered as “one size fits all.” This makes clear the huge challenge in changing behavior. Health awareness campaigns can certainly be useful, but changing the costs and benefits of behavior for all is a far more economically costly and comprehensive project for societies. Improving future prospects – for example, by providing good educational and job opportunities, removing discrimination, and reducing risks in the social environment, such as crime – are all likely to encourage individuals to favor a longer-term view and hence may promote health-preserving behavior, especially in disadvantaged groups.

## Looking ahead

Our goal has been to imagine a future in which evolutionary medicine accelerates biomedical solutions and informs effective public health policies. As we all face an increasingly uncertain future, the effective application of evolutionary medicine’s many tools and lenses (including life-history theory, trade-offs, coevolution, mismatch, constraints on selection, multi-level selection, and sexual selection) (4) will be crucial in helping counter emerging health challenges. Evolutionary medicine has already yielded valuable insights in fields including cancer, infectious diseases, and reproductive health, using frameworks based on the emergence of resistance and life-history theory, but there is far greater potential yet to be realized.

A broad range of predictions and models informed by evolutionary principles can be harnessed to support researchers working to counter the adverse health effects anticipated in the century to come. We have outlined some of the most important areas in which evolutionary approaches are being used to develop more effective strategies for a range of human health concerns. We have shown how evolutionary medicine’s profoundly integrative approach connecting health across generations, species, and ecosystems can reveal insights otherwise obscured by traditionally reductive perspectives.

As the geneticist and evolutionary biologist Theodosius Dobzhansky asserted, “Nothing in biology makes sense except in the light of evolution” (174) and in turn evolutionary biology shapes all aspects of life on Earth. Therefore, all life science innovation in the coming decades will rely to a significant degree on approaches now being developed by leaders in evolutionary medicine. Greater interdisciplinary collaboration is vital to systematically leverage the insight-generating power of evolutionary medicine to understand, prevent, and treat existing and emerging threats to human, animal, and planetary health.

## Data Availability

The original contributions presented in the study are included in the article/supplementary material. Further inquiries can be directed to the corresponding authors.

## Key points

- Evolutionary medicine can apply insights from evolution and ecology to spark transformational innovation in biomedicine and inform effective health policies.
- Systematic mapping is required to identify animal model systems for disease vulnerability, resistance, and counter-resistance that could lead to novel clinical treatments.
- Evolutionary dynamics should also guide the development of novel therapeutic approaches that target the development of treatment resistance in cancers and antimicrobial resistance.
- Many modern human diseases result from mismatches between the ecologies in which we evolved and our modern environments, while others reflect life-history evolution – these insights have important implications for public health policies and disease-prevention efforts.
- Effective management of the COVID-19 (SARS-CoV-2) pandemic and future zoonotic outbreaks requires applying evolutionary theories to understand and model infection dynamics and create models of future scenarios.
- Evolutionary principles can also be used to understand and address aspects of human behavior that impede biomedical innovation and public health (e.g., vaccine hesitancy).

## BOX 1 Evolutionary medicine offers numerous opportunities to understand disease causation, improve disease prevention, and accelerate biomedical innovation – we focus on the aspects summarized here.

**Table:**

	Disease causation	Disease prevention	Biomedical innovation
Role and opportunity	Better understand the etiology of diseases Forecast future disease burdens	Inform public health policy and disease prevention efforts	Accelerate transformational therapeutic innovation
Examples	Evolutionary and developmental mismatches, e.g. • obesity, type 2 diabetes, cardiovascular disease • allergy • microbiome change • mental health Infectious disease epidemiology, e.g. • evolution in virulence and transmissibility (including COVID-19/SARS-CoV-2 and future pandemics) Life history trait evolution • growth, reproduction, and survivorship dynamics Human behavior, e.g. • inclusive fitness vs survival trade-offs		Cancer, e.g., targeting cancer resistance evolution • adaptive approaches • extinction approaches
			Infectious diseases, e.g., targeting antimicrobial resistance evolution • anti-antibiotics • phage therapy

## BOX 2 Selected representative research priorities in evolutionary medicine to address high-impact human health and public health challenges.

- A grand challenge to develop phylogenetically-informed databases as resources for identifying novel animal models of resilience and vulnerability to disease
- Development of evolution-resistant chemotherapies for cancer and pathogen-induced diseases
- Clinical trials leveraging phage therapy to reduce pathogen virulence and avoid resistance (e.g., for multiple-drug-resistant bacterial infections, chronic otitis, and venous ulcers)
- Identification of, and health interventions for, highest-risk individuals for chronic diseases linked to life-history patterns, e.g., type 2 diabetes and breast cancer risks, which are modified by pregnancy and breastfeeding
- Feasibility and efficacy of population-level reductions in lifestyle-related diseases (e.g., atherosclerosis, metabolic dysfunction, obesity) through gestational, perinatal, and early-life investments
- Comparative research to understand the conditions under which transmissibility reduces virulence and applying this to shape more effective early response to future pandemic threats
- Large-scale behavioral intervention studies informed by kin selection, human heterogeneity, and ecological variation (e.g., to address vaccine hesitancy, increase exercise, and reduce stigma-associated barriers to treatment reluctance)
